#  Comparing the Fracture Rate of Hero 642, FlexMaster and Mtwo in the Simulated Canals

**Published:** 2014-03-08

**Authors:** Hosein Labaf, Roza Haghgoo, Kiumars Nazarimoghadam, Mahshid Mohamadibasir

**Affiliations:** a * Department of Endodontics, Dental School, Shahed University, Tehran, Iran;*; b * Department of Pediatric Dentistry, Dental School, Shahed University, Tehran, Iran; *; c *Department of Restorative Dentistry, Dental School, Shahed University, Tehran, Iran*

**Keywords:** Dental Instruments, Fatigue Fracture, Nickel-Titanium Alloy, Root Canal Preparation, Torsional Force

## Abstract

**Introduction:** File fracture is one of the main procedural mishaps in endodontic treatment. The aim of this *in vitro* study was to compare the fracture rate of three NiTi rotary systems; Hero 642, Mtwo and FlexMaster in artificial canals. **Methods and Materials:** In this study, bovine long bone was used. After primary preparation of bones, longitudinal sections with 4-cm diameter were cut and encoded. Subsequently, semicircular sections were prepared. A total number of 500 canals were created in the same way; the upper 3 mm of the canals were initially prepared with orifice shapers and then canals were filed with FlexMaster files sizes 25/0.02 and 25/0.04 to 13 mm of canal length. The prepared canals were assigned into 3 groups of the following systems: Hero 642, Mtwo and FlexMaster. Six selected instruments were used from each system; the files were applied 13 mm along the canals for 10 sec with manufacturer’s suggested speed and torque. The number of the canals prepared by each file before its separation was recorded; finally the data was analyzed with ANOVA test. **Results:** Mean number of prepared canals in Mtwo, FlexMaster and Hero groups before file separation was 15, 25 and 32, respectively. **Conclusion: **Results of this study showed that the number of prepared canals by Hero 642 was more than FlexMaster and Mtwo systems.

## Introduction

Root canal preparation is one of the main steps in endodontic practice [1]. One of the main objectives of root canal preparation is to create continuously tapered and conical form whilst maintaining the original shape of the canal [2-4]. Creating a safe and tapered shape of the canal with stainless steel instruments is not as easy as canal preparation with nickel–titanium (NiTi) rotary instruments [2, 5-8]. Super elasticity of NiTi rotary files have facilitated root canal preparation even in curved canals [1, 9, 10], and it is suggested that the adoption of NiTi rotary instrumentation would improve the cleaning and shaping of root canals and the quality of the root-filling [11, 12].

Although the NiTi instruments are more flexible than the stainless steel files, the main problem is the failure of the instruments due to excessive torsion and flexural stress [13-15]. Mechanical properties of NiTi instruments are influenced by factors such as cross-section, flute design, raw material, and manufacturing processes [16, 17].

Several factors may affect the fracture rate of rotary instrument such as shape of the canal [18], rotational speed [19, 20], number of uses [1] and cross sectional design of the file [21, 22]. Cross sectional area (core) and its design may influence the resistance of instrument as well; instruments with larger diameters have greater accumulation of internal stress [23]. This relationship is not always true, meaning that an increase in instrument diameter may cause its increased resistance to fracture [24, 25]. In a study for determining the stiffness of ProFile (Maillefer, Ballaigues, Switzerland) and Hero Shaper (Micro-Mega, Besancon, France) with similar triangular cross sections, Mtwo (VDW, Munich, Germany) with an S-shaped rectangle-based design and NRT (Mani Inc., Shioya-gun, Japan) with a modified rectangle-based design, it was defined that NiTi instruments with rectangular cross sections created higher stress differentials during simulated canal shaping and may encounter higher residual stress and plastic deformation than instruments with triangle-based cross sections [21].

**Table 1:** Mean number of canals prepared with FlexMaster, Mtwo and Hero 642

**Instrument type**	**Number**							**Mean (SD)**
**FlexMaster**	File	1	2	3	4	5	6	15.3 (2.4)
	Treated canals	13	17	13	19	16	14	
**M** **two**	File	1	2	3	4	5	6	23.3 (3.98)
	Treated canals	24	24	21	23	32	28	
**Hero 642**	File	1	2	3	4	5	6	32.3 (6.6)
	Treated canals	37	40	29	33	21	33	

**Table 2:** Mean (SD) fracture rate based on file type (mm)

**N**	**FlexMaster**	**Mtwo**	**Hero 642**
**1**	4.5	7.0	4.5
**2**	3.5	5.0	6.5
**3**	5.5	6.0	3.0
**4**	6.0	7.0	5.5
**5**	2.0	7.0	4.5
**6**	2.0	6.0	4.5
	3.92(1.71)	6.33(0.82)	4.75(1.17)

One study measured the cyclic fatigue of ProTaper (Dentsply Maillefer, Ballaigues, Switzerland), FlexMaster (Vereinigte, Dentalwerke, Munich, Germany), Mtwo and ProFile; the results showed that Mtwo had the highest resistance to fatigue compared to the other instruments [18]. In another study, torsion and bending properties of six NiTi instruments with different cross-section designs [ProTaper, Hero 642, Mtwo, ProFile, Quantec (Tycom, Irvine, CA, USA), and NiTi Flex hand file (Maillefer, Ballaigues, Switzerland)] were measured and the results revealed that ProTaper and Hero 642 showed the lowest stress levels making them the most resistant while the NiTi-Flex model was the weakest [22].

This study was designed considering several factors affecting the separation of rotary instruments aiming at comparing the fracture rate of FlexMaster, Hero 642 and Mtwo in artificial canals.

## Methods and Materials

In this *in vitro* study, bovine long bone was used because of its similar physical and chemical structure to dentin [26, 27]. After primary preparation of bones (using 5.25% NaOCl for 10 min), longitudinal sections with 4 cm diameter were cut and encoded. Then semicircular sections with 6 mm radius were prepared in longitudinal sections using a 12-mm diameter trephine bur (KLS Martin Group, Gebrüder Martin GmbH & Co. KG, Germany); five hundred canals were made in the same manner. All The canals were prepared initially with the orifice shaper, Introfile (VDW, Munich, Germany), with a 3-mm length and then with FlexMaster (Vereinigte, Dentalwerke, Munich, Germany) sizes 25/0.02 and 25/0.04, in 13-mm working length. Then all the canals were assigned into 3 groups (for matching the samples, 3 types of the files were used on each bone section). In each group, six selected files of each system [Hero 642 (Micro-Mega, Besancon, France) size 25/0.06, Mtwo (VDW, Munich, Germany) size 25/0.06 and FlexMaster (Vereinigte, Dentalwerke, Munich, Germany) size 25/0.06, all with 25-mm lengths, were used. Each file was applied in canals that were 13-mm long with recommended speed and torque using a torque controller system, Endo IT professional (Aseptico Inc., Woodinville, WA, USA), for 10 sec (choosing this time period, was based on a pilot study) by a trained dentist. The total time for each file before separation was 60 sec. Canal lubricant (RC-Prep, Premier Dental Products, Philadelphia, USA) was used for better filing and clinical resemblance. The number of canals prepared by a file before its fracture was recorded, and finally the data was analyzed with ANOVA and LSD tests. The level of significance was set at 0.05.

## Results

Mean number of canals prepared by 3 types of files, are presented in Table 1. ANOVA and LSD tests showed a significant difference in number of prepared canals in 3 groups (*P*=6.58). Difference in rate of file fracture between Mtwo and two other groups (Hero 642 and FlexMaster) was statistically significant while the difference between FlexMaster and Hero 642 was not significant (Table 2).

## Discussion

Root canal preparation consists of cleaning and shaping of the canal. Due to their superelasticity, NiTi instruments have facilitated the preparation of curved root canals [1, 8, 12, 17]. However instrument fracture in rotary motion is still a concern [28]. The purpose of this study was to compare fracture rate of Mtwo, FlexMaster and Hero 642 in the artificial canals. We chose simulated canals in order to standardize the groups. For each system, the similar sizes of instruments were chosen.

Results of this study showed that the number of prepared canals before separation by Hero 642 is higher compared to FlexMaster and Mtwo. It can be assumed that Hero 642 has triangle cross-section and its central core is wide compared to Mtwo and FlexMaster; so it is more resistant to fracture [29]. Stability of pitch length in Hero 642 and flaring of its central core causes this file to be fracture resistant compared to Mtwo and FlexMaster.

Kim *et al.* found that NiTi instruments with S-shaped cross-section designs such as Mtwo, create higher stress than instrument with triangular shaped cross sections like Hero 642 [21]. This finding is in agreement with our results. Results of the study by Sun *et al*. showed that the elapsed time before the fracture of Mtwo was significantly more than Hero 642 [30]. The difference between the results of these two studies can be related to the different study designs. In our study, artificial canals were prepared from bovine bone due to its similarity to human dentin.

In this study, each canal was used only one time and the high number of canals (a total number of 500) approximate our study to clinical conditions. Sample matching was similar to other studies [31], however for exact sample distribution, 3 types of files were used on each piece of bone. In metal samples, tensile forces, remain in the file in the form of fatigue stress that doesn't simulate clinical condition. This can justify the results of some studies not being significant [32]. The influence of the force exerted on the file by operator is an important factor [33, 34].

In this study, experimental files were applied with minimum force. We pre-flared the canals with an orifice shaper (Introfile), to reduce the time of applying rotary instruments and to decrease the stress exerted on them. Pre-flaring can have an effect on maintaining the original path of the canal during rotary instrumentation [35]. One limitation of this study was conducting the instrumentation on simulated canals. Although bovine bone simulates the hardness of dentine, the findings cannot be directly transposed to clinical practice, mainly because of the circular cross section of simulated canals, which will influence the torsion load of instruments. 

In this study, the special size of canals was compared to each other. Because of importance of alloy and design of files, evaluating the effects of these two factors is suggested for the future studies.

## Conclusions

From the results of the current study it can be concluded that although the rate of fracture in Mtwo was significantly higher than two other systems (*i.e.* FlexMaster and Hero 642), but a rather high number of canals were prepared with Mtwo files (an average of 25 for each file) and this fact is considered clinically important.
